# Surveillance of rhinovirus diversity among a university community identifies multiple types from all three species including an unassigned rhinovirus A genotype

**DOI:** 10.1111/irv.13057

**Published:** 2022-09-28

**Authors:** Temitope O. C. Faleye, Amir Elyaderani, Peter Skidmore, Sangeet Adhikari, Abriana Smith, Nicole Kaiser, Helen Sandrolini, Sarah Finnerty, Rolf U. Halden, Arvind Varsani, Matthew Scotch

**Affiliations:** ^1^ Biodesign Center for Environmental Health Engineering, Biodesign Institute Arizona State University Tempe Arizona USA; ^2^ College of Health Solutions Arizona State University Tempe Arizona USA; ^3^ School of Sustainable Engineering and the Built Environment Arizona State University Tempe Arizona USA; ^4^ Health Services Arizona State University Tempe Arizona USA; ^5^ OneWaterOneHealth Nonprofit Project of the Arizona State University Foundation Tempe Arizona USA; ^6^ Biodesign Center for Fundamental and Applied Microbiomics, Center for Evolution and Medicine, School of Life Sciences Arizona State University Tempe Arizona USA

**Keywords:** high‐throughput nucleotide sequencing, population surveillance, rhinovirus, Southwestern United States, student health services

## Abstract

We determine the presence and diversity of rhinoviruses in nasopharyngeal swab samples from 248 individuals who presented with influenza‐like illness (ILI) at a university clinic in the Southwest United States between October 1, 2020 and March 31, 2021. We identify at least 13 rhinovirus genotypes (A11, A22, A23, A25, A67, A101, B6, B79, C1, C17, C36, and C56, as well a new genotype [AZ88**]) and 16 variants that contributed to the burden of ILI in the community. We also describe the complete capsid protein gene of a member (AZ88**) of an unassigned rhinovirus A genotype.

## 1 INTRODUCTION

In the United States, the annual economic burden of respiratory tract infections caused by viruses is in the order of ~$50 billion.^1^, ^2^ Rhinoviruses are one of several virus groups that cause respiratory tract infections and have been implicated in the etiology of about 50% of asthma and chronic obstructive pulmonary disease exacerbations.^3^

Rhinoviruses consist of over 160 genotypes grouped into three species (*Rhinovirus A, Rhinovirus B, and Rhinovirus C*) in genus *Enterovirus*, family *Picornaviridae*. The positive‐sense, single‐stranded, ~7.2 kb RNA genome of rhinoviruses encodes a large polyprotein that is autocatalytically cleaved into 11 proteins; four (VP1–VP4) and seven (2A–2C, 3A–3D) structural (or capsid) and nonstructural proteins, respectively.^3^ Rhinoviruses were originally classified into types using neutralization assays.^4^ Recently (as with other enteroviruses), classification (and type assignment) is done using pairwise identity of nucleotide sequences that represent complete genomes, complete capsid protein, or VP1 genes.^5^ Specifically, rhinovirus type demarcation criteria stipulate that members of the same type be around 11% divergent in the VP1 capsid protein gene.^4^, ^6^

## 2 METHODS

As part of ongoing respiratory virus surveillance project, we received 248 nasopharyngeal swab samples from patients presenting with influenza‐like illness (ILI) at a university health clinic in the Southwest United States between October 1, 2020 and March 31, 2021. We pooled the samples by month by taking 20 μl aliquots from each sample. Pools that exceeded 500 μl were concentrated to 500 μl using 10,000 MW cutoff centrifugal filters (MilliporeSigma, USA). Subsequently, 140 μl of each pool was subjected to RNA extraction using the QiaAmp RNA extraction kit (Qiagen, Germantown, MD, USA) following manufacturer's instructions. The RNA extract was then subjected to two pan‐enterovirus complete capsid RT‐PCR assays (amplicon size of ~3900 bp, volume 25 μl each) as previously described in Faleye et al.^7^ The PCR products were pooled, and 2 μl of the pooled PCR product was used as template for a second pan‐enterovirus partial (~350 bp) VP1 PCR assay as previously described in Faleye et al.^7^ The ~350 bp partial VP1 products were Sanger sequenced using both the forward and reverse primers. Also, 20 μl of the remaining 48 μl (complete capsid protein gene assay product) was cleaned and used for library preparation (KAPA Hyperplus Library Kit) and paired‐end sequenced (2 × 250 bp) on an Illumina MiSeq sequencer (Illumina, San Diego, CA, USA) at the Biodesign Institute, Arizona State University.

The Sanger sequencing data were typed using a BLASTn search against the GenBank nucleotide database. For the Illumina sequencing derived data, we used the KBase platform to trim the raw reads with Trimmomatic v0.36, de novo assemble using MEGAHIT v1.2.9 and determine depth of coverage and total mapped reads with Bowtie2 v2.3.2., all using default parameters.^8^ Viral contigs (>2000 bp, mean coverage >50x) were identified using both the enterovirus genotyping tool (EGT) and a BLASTn search of the GenBank nucleotide database. Pairwise identity analysis was done using SDT v1.2.^9^

For phylogenetic analysis, the rhinovirus contigs assembled from Illumina raw reads were used as query in a BLASTn search of the GenBank nucleotide database, and the top five hits per contig were downloaded. This was assembled into a local database, and duplicate hits were removed. Rhinovirus contigs assembled from the Illumina raw reads generated in this study were added to our local sequence database which was then subjected to multiple sequence alignments (MSA) using ClustalW in MEGAX.^10^ Subsequently, a maximum likelihood (ML) phylogenetic tree was inferred in MEGAX with GTR substitution model and 1000 bootstrap replicates.

## 3 RESULTS

The ~350 bp amplicon was detected in all six samples (Figure S1) suggesting that both the ~3900 and ~350 bp assays worked, and all samples contained enteroviruses. It is important to mention that one sample (February 2021) had two very distinct bands, while another (November 2020) had smears around 350 bp suggesting more than one EV type was present per sample. This was confirmed using Sanger sequencing (multiple peaks) of the ~350 bp product (Table 1).

**TABLE 1:** Summary data from Sanger and Illumina sequencing of rhinovirus amplicons

			Sanger sequencing of partial VP1	Illumina sequencing of complete capsid				
S/N	Month‐year	Samples per pool	EV types	EV types	Length (nt)	Mean coverage (x)	Total mapped reads (%)	Total reads
1	Oct‐2020	44	RV‐A11	RV‐A11	2587	3047	40,561 (3.29)	1,233,542
				RV‐A22	4774	276		
2	Nov‐2020	64	MP	RV‐A25	4875	4923	1,511,512 (84.97)	1,778,962
				RV‐A67	5204	3273		
				RV‐AZ88**	3996	357		
				RV‐A101	4588	1288		
				RV‐C56	5725	50,922		
3	Dec‐2020	31	RV‐B79	RV‐B79	6080	261,890	7,087,310 (70.81)	10,008,578
				RV‐B6	5320	3090		
				RV‐C56	2617	1743		
4	Jan‐2021	41	RV‐C1	RV‐C1	4768	4634	105,337 (4.72)	2,233,142
5	Feb‐2021	48	MP	RV‐B79	6375	6694	938,334 (82.97%)	1,130,904
				RV‐C17	2726	60		
				RV‐C1	3551	2490		
				RV‐C36	2069	79 677		
6	Mar‐2021	20	RV‐A23	RV‐A23	5342	4032x	102,000 (4.95)	2,060,452

*Note*: Method: Trimmomatic‐v0.36, MEGAHIT v1.2.9, Bowtie2‐v2.3.2 in KBase (Contig length >2 kb, coverage >50x).

In Table 1, we see that there was consistent agreement between Sanger and high‐throughput sequencing methods for determination of rhinovirus types. Samples for which Sanger sequencing data suggested a specific virus type were confirmed as the dominant type (based on mean coverage depth) in the subsequent Illumina data. However, Illumina also showed that other rhinovirus types were potentially present, possibly at lower concentration (considering the number of mapped reads). In addition, in the instance of multiple peaks (November 2020 and February 2021) in the Sanger data, the Illumina data confirmed that more than one rhinovirus type was present.

**FIGURE 1:**
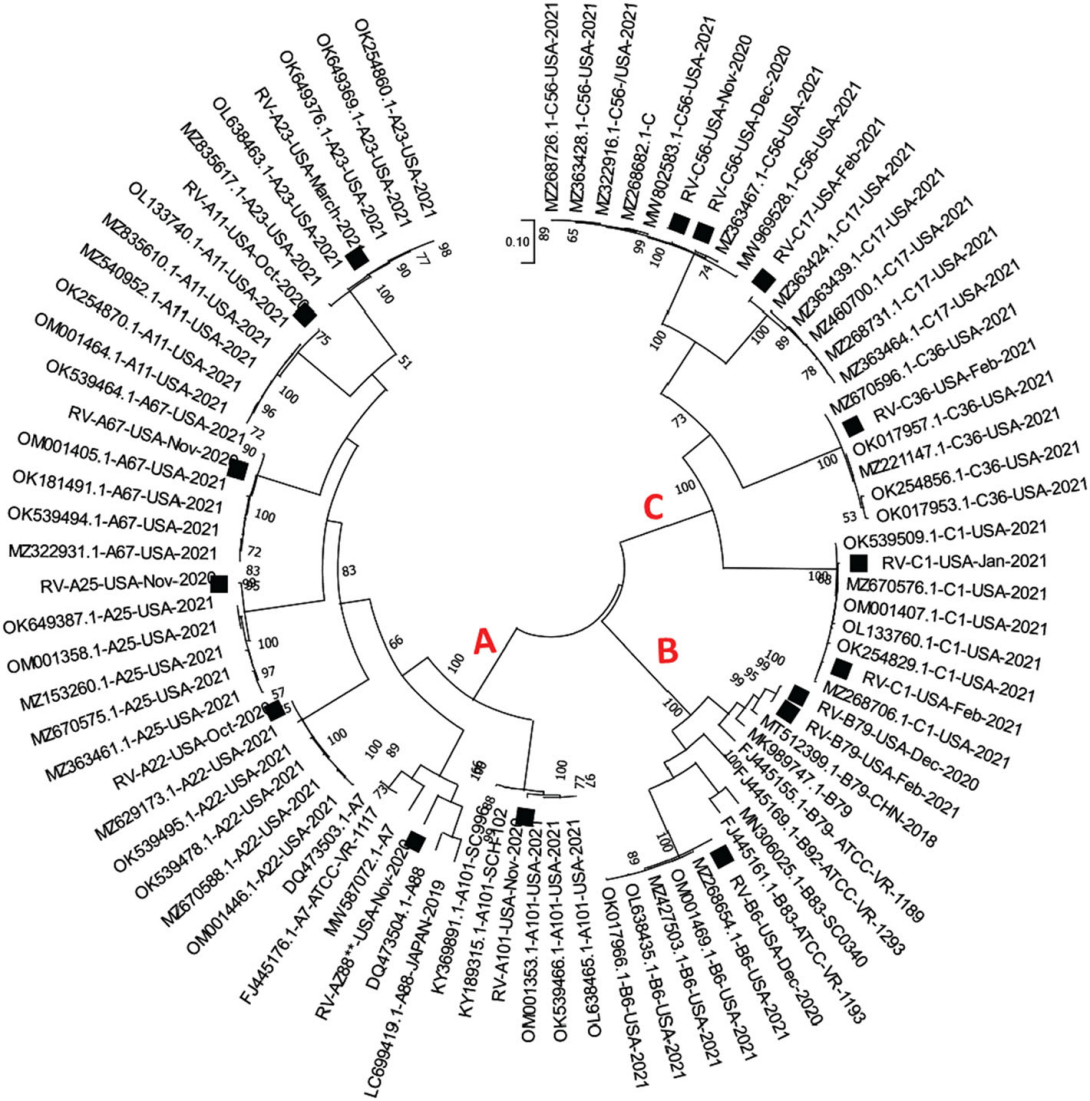
Maximum‐likelihood tree of rhinovirus sequences recovered in this study (black squares) and their top five hits in GenBank. The tree was inferred using the VP3 genomic region. Bootstrap values are indicated if greater than 50%. The letters, A, B, and C represent the three different rhinovirus species.

We identified 13 rhinovirus types (A11, A22, A23, A25, A67, A101, B6, B79, C1, C17, C36, and C56 as well as a new genotype for sequence AZ88) and 16 variants (Table 2) from all three rhinovirus species that circulated in the university community during the season. The number of distinct rhinovirus types circulating per month ranged from one to five (Table 1). Some types (B79, C1, and C56) circulated for more than a month (Table 1). BLASTn (Table 3) and phylogenetic analysis (Figure 1) showed that the rhinovirus types recovered on the university campus (except for AZ88 and B79) were representative of variants recovered from other parts of the United States in early 2021. While the B79s were 94% similar to their closest sequence in GenBank, AZ88 was 84.02% to 84.13% similar to its most similar sequence types in GenBank (Table 3). As previously mentioned, this is greater than the accepted divergence threshold (of around 11%) for members of individual rhinovirus types.^4^

**TABLE 2:** HRV types and variants detected in NP swabs of individuals presenting with ILI to the university clinic in SW USA from October 2020 to March 2021

RV species	Types present in table above	Total types	Total variants
A	11, 22, 23, 25, 67, AZ88**, 101	7	7
B	6, 79 (*n* = 2)	2	3
C	1 (*n* = 2), 17, 36, 56 (*n* = 2)	4	6
**Total**		13	16

Abbreviation: ILI, influenza‐like illness.

**TABLE 3:** Results of BLASTn search of the GenBank database showing pairwise identity of HRV variants detected in this study and their most similar sequences in GenBank

	ID	Accession #	Rhinovirus type	Accession #	Pairwise identity %	Country/year	Accession #	Pairwise identity %	Country/year
1	RV‐A11‐USA‐Oct‐2020	ON989181	A11	MZ835610	99.07	USA‐2021	OL133740	99.03	USA‐2021
2	RV‐A22‐USA‐Oct‐2020	ON989182	A22	OM001446	99.46	USA‐2021	OK539478	99.46	USA‐2021
3	RV‐A25‐USA‐Nov‐2020	ON989183	A25	MZ153260	99.24	USA‐2021	MZ363461	98.95	USA‐2021
4	RV‐A67‐USA‐Nov‐2020	ON989184	A67	OK539464	99.58	USA‐2021	MZ322931	98.79	USA‐2021
5	RV‐AZ88**‐USA‐Nov‐2020	ON989185	AZ88**	LC699419	84.13	JAPAN‐2019	MW587072	84.02	China‐2016/2017
6	RV‐A101‐USA‐Nov‐2020	ON989193	A101	KY189315	98.61	USA‐2016	KY369891	98.61	USA‐2016
7	RV‐C56‐USA‐Nov‐2020	ON989186	C56	MW969528	99.02	USA‐2021	MZ268682	98.95	USA‐2021
8	RV‐B79‐USA‐Dec‐2020	ON989194	B79	MT512399	96.35	CHINA‐2018	MK989747	94.06	Kenya‐2010
9	RV‐B6‐USA‐Dec‐2020	ON989187	B6	MZ427503	99.59	USA‐2021	MZ268654	99.59	USA‐2021
10	RV‐C56‐USA‐Dec‐2020	ON989195	C56	MZ363467	99.50	USA‐2021	MZ363428	99.16	USA‐2021
11	RV‐C1‐USA‐Jan‐2021	ON989188	C1	MZ268706	99.10	USA‐2021	MZ670576	99.06	USA‐2021
12	RV‐B79‐USA‐Feb‐2021	ON989189	B79	MT512399	96.33	CHINA‐2018	MK989747	94.16	Kenya‐2010
13	RV‐C17‐USA‐Feb‐2021	ON989190	C17	MZ363439	97.58	USA‐2021	MZ363424	97.54	USA‐2021
14	RV‐C1‐USA‐Feb‐2021	ON989196	C1	MZ268706	99.72	USA‐2021	OM001407	99.63	USA‐2021
15	RV‐C36‐USA‐Feb‐2021	ON989191	C36	MZ670596	99.52	USA‐2021	OK017957	99.32	USA‐2021
16	RV‐A23‐USA‐March‐2021	ON989192	A23	OK649376	99.68	USA‐2021	OK649369	99.68	USA‐2021

We further explored this finding by extracting the complete VP1 region of the putative new genotype for sequence AZ88 contig (subsequently referred to as AZ88**) and used it in a BLASTn search of the GenBank database to identify and download the top 20 hits. We aligned these sequences alongside the AZ88** VP1 (two sequences [KP737112 and KP737113] were removed because they did not have complete VP1) and reference sequences of RV‐A7, A36, A58, A88, and A89 (which have been shown to all form a distinct cluster).^6^ We used the alignment to infer a maximum likelihood tree, as described above, and pairwise similarity analysis using SDT v1.2.^9^

**FIGURE 2:**
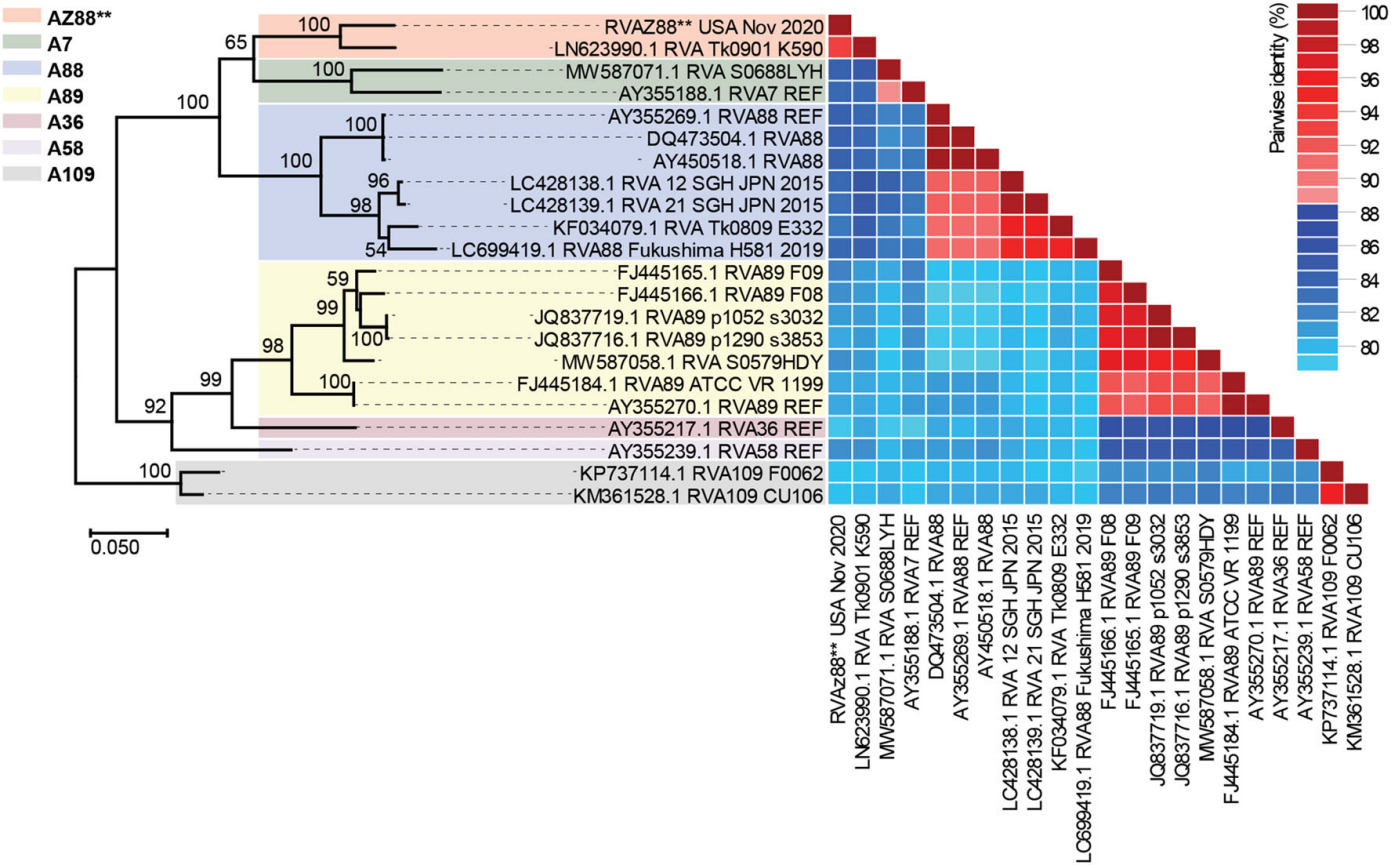
Genetic characterization and similarity of rhinoviruses. Maximum‐likelihood tree and pairwise identity analysis of AZ88** VP1, its top 20 BLASTn hits and reference sequences of rhinovirus A7, A36, A58, A88, A89, and A109. Bootstrap values are indicated if greater than 50%. Pairwise identity above and below 88% are colored red and blue, respectively. Please see Table S1 for the pairwise identity data that were used to make the matrix in this figure.

The results (Figure 2) show that AZ88** is not a member of RV‐A88 but rather is most similar to LN623990 (pairwise identity = 93.26%, Table S1), a novel (yet to be numbered) Rhinovirus A member recovered from a case of acute lower respiratory tract illness in Cambodia in 2009.^11^ The fact that the complete capsid or genome of LN623990 was not sequenced explains why it did not emerge in the top hits during our initial BLASTn search using the complete contig of AZ88**. Hence, it seems that AZ88** is another member of this unnumbered Rhinovirus A type and the first complete capsid described globally based on publicly available data in GenBank as of July 2022.

## 4 DISCUSSION AND CONCLUSION

In this study, we identify at least 13 rhinovirus types and 16 variants (Table 2) from all three rhinovirus species that circulated in the university community between October 1, 2020 and March 31, 2021 and appear to have contributed to the burden of ILI in the community during the SARS‐CoV‐2 pandemic. Circulation of these rhinovirus types (except AZ88** and B79) in the United States in 2021 was independently confirmed (Table 3 and Figure 1). We also describe the first complete capsid protein gene sequence of a novel rhinovirus A genotype that is yet to be assigned a number and for which only one VP1 sequence has been described since 2009 (based on publicly available sequence data in GenBank as of July, 2022). Furthermore, though originally designed to amplify complete capsid protein sequence region of poliovirus (an *Enterovirus C*. species member),^12^ coupled with the findings of this study, we and others have now shown that the PanEV RT‐PCR assay can amplify the same region from other enterovirus species (particularly enterovirus species A, B, C, D, and G and rhinovirus species A, B, and C) and canine picornavirus genomes.^13^, ^14^, ^15^

We are aware that the diversity of rhinovirus types described here might not be a complete catalog of all the types present in the samples screened or that circulated in the university community during the period sampled. Particularly, the fact that we pooled only 20 μl of each resuspended NP swab sample coupled with the stringent (>2000 nt contig length and >50x coverage; Table 1) cutoff used in our assembly and detection pipeline could have prevented detection of low titer rhinovirus (or other enterovirus) types present in the samples. Furthermore, the varying length of contigs recovered suggests that the reverse primer might have initiated cDNA synthesis at different genomic regions on the respective genomes detected in a manner that is neither species specific nor *cre*‐dependent. Further work is therefore necessary to better define and establish the breadth of the pan‐enterovirus complete capsid RT‐PCR assay.

## CONFLICT OF INTEREST

R.U.H. is the founder of OneWaterOneHealth, a non‐profit project of the Arizona State University Foundation.

## AUTHOR CONTRIBUTIONS

**Temitope O.C. Faleye:** Conceptualization; data curation; formal analysis; investigation; methodology; validation; visualization. **Amir Elyaderani:** Methodology. **Peter Skidmore:** Methodology. **Sangeet Adhikari:** Methodology. **Abriana Smith:** Methodology. **Nicole Kaiser:** Methodology. **Helen Sandrolini:** Resources. **Sarah Finnerty:** Resources. **Rolf U. Halden:** Funding acquisition; resources; supervision. **Arvind Varsani:** Funding acquisition; resources; supervision. **Matthew Scotch:** Funding acquisition; resources; supervision.

### PEER REVIEW

The peer review history for this article is available at https://publons.com/publon/10.1111/irv.13057.

## Supporting information


**Table S1.** **Pairwise identity and distance of sequences in Figure 2**.

**Figure S1.** **Gel electrophoresis result of partial VP1 (~350 bp) assay**. Lanes 1 and 8 are 100 bp molecular ladder. Gel visualized using BioRad Gel Doc XR + system running Image lab 4.1 software with option to “highlight saturated pixels” enabled.

## Data Availability

The sequences described in this study have been deposited in GenBank under accession numbers ON989181‐ON989196.
