# Supplementary material for: Surveillance of rhinovirus diversity among a university community identifies multiple types from all three species including an unassigned rhinovirus A genotype
Source: Influenza Other Respir Viruses. 2022 Sep 28;17(1):e13057. doi: 10.1111/irv.13057 (PMC9835438; doi:10.1111/irv.13057)
Supplement: Supplementary file 1 — Table S1. Pairwise identity and distance of sequences in Figure 2. [file IRV-17-e13057-s002.docx]

**Appendices**

**Table S1.** **Pairwise identity and distance of sequences in Figure 2**.

| **First Sequence** | **Second Sequence** | **Pairwise Identity %** |
| --- | --- | --- |
| AY355188_RVA7_REF | AY355217_RVA36_REF | 78.67 |
| DQ473504_RVA88 | FJ445165_RVA89_F09 | 78.86 |
| DQ473504_RVA88 | MW587058_RVA_S0579HDY | 78.86 |
| AY355269_RVA88_REF | FJ445165_RVA89_F09 | 78.86 |
| AY355269_RVA88_REF | MW587058_RVA_S0579HDY | 78.86 |
| AY450518_RVA88 | FJ445165_RVA89_F09 | 78.86 |
| AY450518_RVA88 | MW587058_RVA_S0579HDY | 78.86 |
| RV-AZ88**-USA-Nov-2020 | AY355217_RVA36_REF | 78.98 |
| DQ473504_RVA88 | JQ837716_RVA89_p1290_s3853 | 78.98 |
| AY355269_RVA88_REF | JQ837716_RVA89_p1290_s3853 | 78.98 |
| AY450518_RVA88 | JQ837716_RVA89_p1290_s3853 | 78.98 |
| DQ473504_RVA88 | JQ837719_RVA89_p1052_s3032 | 79.09 |
| AY355269_RVA88_REF | JQ837719_RVA89_p1052_s3032 | 79.09 |
| AY450518_RVA88 | JQ837719_RVA89_p1052_s3032 | 79.09 |
| LC428139_RVA_21_SGH_JPN_2015 | FJ445165_RVA89_F09 | 79.09 |
| MW587071_RVA_S0688LYH | AY355217_RVA36_REF | 79.28 |
| LC428139_RVA_21_SGH_JPN_2015 | MW587058_RVA_S0579HDY | 79.33 |
| LC699419_RVA88_Fukushima_H581_2019 | KP737114_RVA_F0062 | 79.33 |
| LN623990_RVA_Tk0901_K590 | KP737114_RVA_F0062 | 79.56 |
| AY355188_RVA7_REF | KP737114_RVA_F0062 | 79.56 |
| LC428139_RVA_21_SGH_JPN_2015 | FJ445166_RVA89_F08 | 79.56 |
| LC428139_RVA_21_SGH_JPN_2015 | KP737114_RVA_F0062 | 79.56 |
| LC699419_RVA88_Fukushima_H581_2019 | KM361528_RVA_CU106 | 79.56 |
| RV-AZ88**-USA-Nov-2020 | KP737114_RVA_F0062 | 79.67 |
| AY355188_RVA7_REF | KM361528_RVA_CU106 | 79.67 |
| LC428138_RVA_12_SGH_JPN_2015 | FJ445165_RVA89_F09 | 79.67 |
| LC428139_RVA_21_SGH_JPN_2015 | JQ837716_RVA89_p1290_s3853 | 79.67 |
| KF034079_RVA_Tk0809_E332 | KP737114_RVA_F0062 | 79.67 |
| LC699419_RVA88_Fukushima_H581_2019 | FJ445184_RVA89_ATCC_VR_1199 | 79.67 |
| LC699419_RVA88_Fukushima_H581_2019 | AY355270_RVA89_REF | 79.67 |
| LC699419_RVA88_Fukushima_H581_2019 | AY355217_RVA36_REF | 79.67 |
| RV-AZ88**-USA-Nov-2020 | KM361528_RVA_CU106 | 79.79 |
| DQ473504_RVA88 | FJ445166_RVA89_F08 | 79.79 |
| AY355269_RVA88_REF | FJ445166_RVA89_F08 | 79.79 |
| AY450518_RVA88 | FJ445166_RVA89_F08 | 79.79 |
| LC428139_RVA_21_SGH_JPN_2015 | JQ837719_RVA89_p1052_s3032 | 79.79 |
| LC428139_RVA_21_SGH_JPN_2015 | FJ445184_RVA89_ATCC_VR_1199 | 79.79 |
| LC428139_RVA_21_SGH_JPN_2015 | AY355270_RVA89_REF | 79.79 |
| LC699419_RVA88_Fukushima_H581_2019 | AY355239_RVA58_REF | 79.79 |
| MW587071_RVA_S0688LYH | JQ837716_RVA89_p1290_s3853 | 79.86 |
| MW587071_RVA_S0688LYH | MW587058_RVA_S0579HDY | 79.86 |
| MW587071_RVA_S0688LYH | AY355239_RVA58_REF | 79.91 |
| LC428138_RVA_12_SGH_JPN_2015 | MW587058_RVA_S0579HDY | 79.91 |
| LC428138_RVA_12_SGH_JPN_2015 | KP737114_RVA_F0062 | 79.91 |
| KF034079_RVA_Tk0809_E332 | FJ445165_RVA89_F09 | 79.91 |
| KF034079_RVA_Tk0809_E332 | AY355239_RVA58_REF | 79.91 |
| KF034079_RVA_Tk0809_E332 | KM361528_RVA_CU106 | 79.91 |
| MW587071_RVA_S0688LYH | FJ445165_RVA89_F09 | 79.98 |
| MW587071_RVA_S0688LYH | JQ837719_RVA89_p1052_s3032 | 79.98 |
| MW587071_RVA_S0688LYH | FJ445184_RVA89_ATCC_VR_1199 | 79.98 |
| MW587071_RVA_S0688LYH | AY355270_RVA89_REF | 79.98 |
| LC699419_RVA88_Fukushima_H581_2019 | FJ445165_RVA89_F09 | 80.02 |
| LC699419_RVA88_Fukushima_H581_2019 | MW587058_RVA_S0579HDY | 80.02 |
| DQ473504_RVA88 | KP737114_RVA_F0062 | 80.14 |
| AY450518_RVA88 | KP737114_RVA_F0062 | 80.14 |
| LC428138_RVA_12_SGH_JPN_2015 | FJ445166_RVA89_F08 | 80.14 |
| LC428138_RVA_12_SGH_JPN_2015 | FJ445184_RVA89_ATCC_VR_1199 | 80.14 |
| LC428138_RVA_12_SGH_JPN_2015 | AY355270_RVA89_REF | 80.14 |
| LC699419_RVA88_Fukushima_H581_2019 | JQ837716_RVA89_p1290_s3853 | 80.14 |
| MW587071_RVA_S0688LYH | KP737114_RVA_F0062 | 80.26 |
| AY355269_RVA88_REF | KP737114_RVA_F0062 | 80.26 |
| LC428138_RVA_12_SGH_JPN_2015 | JQ837716_RVA89_p1290_s3853 | 80.26 |
| LC428139_RVA_21_SGH_JPN_2015 | AY355239_RVA58_REF | 80.26 |
| KF034079_RVA_Tk0809_E332 | MW587058_RVA_S0579HDY | 80.26 |
| KF034079_RVA_Tk0809_E332 | FJ445184_RVA89_ATCC_VR_1199 | 80.26 |
| KF034079_RVA_Tk0809_E332 | AY355270_RVA89_REF | 80.26 |
| LC699419_RVA88_Fukushima_H581_2019 | FJ445166_RVA89_F08 | 80.26 |
| LC699419_RVA88_Fukushima_H581_2019 | JQ837719_RVA89_p1052_s3032 | 80.26 |
| LN623990_RVA_Tk0901_K590 | KM361528_RVA_CU106 | 80.37 |
| AY355269_RVA88_REF | AY355217_RVA36_REF | 80.37 |
| LC428138_RVA_12_SGH_JPN_2015 | JQ837719_RVA89_p1052_s3032 | 80.37 |
| LC428139_RVA_21_SGH_JPN_2015 | AY355217_RVA36_REF | 80.37 |
| KF034079_RVA_Tk0809_E332 | FJ445166_RVA89_F08 | 80.37 |
| KF034079_RVA_Tk0809_E332 | AY355217_RVA36_REF | 80.37 |
| LN623990_RVA_Tk0901_K590 | AY355217_RVA36_REF | 80.49 |
| DQ473504_RVA88 | AY355217_RVA36_REF | 80.49 |
| AY450518_RVA88 | AY355217_RVA36_REF | 80.49 |
| LC428139_RVA_21_SGH_JPN_2015 | KM361528_RVA_CU106 | 80.49 |
| KF034079_RVA_Tk0809_E332 | JQ837716_RVA89_p1290_s3853 | 80.49 |
| RV-AZ88**-USA-Nov-2020 | FJ445184_RVA89_ATCC_VR_1199 | 80.60 |
| RV-AZ88**-USA-Nov-2020 | AY355270_RVA89_REF | 80.60 |
| LN623990_RVA_Tk0901_K590 | FJ445184_RVA89_ATCC_VR_1199 | 80.60 |
| LN623990_RVA_Tk0901_K590 | AY355270_RVA89_REF | 80.60 |
| DQ473504_RVA88 | KM361528_RVA_CU106 | 80.60 |
| AY450518_RVA88 | KM361528_RVA_CU106 | 80.60 |
| KF034079_RVA_Tk0809_E332 | JQ837719_RVA89_p1052_s3032 | 80.60 |
| MW587071_RVA_S0688LYH | KM361528_RVA_CU106 | 80.72 |
| AY355269_RVA88_REF | KM361528_RVA_CU106 | 80.72 |
| LC428138_RVA_12_SGH_JPN_2015 | AY355217_RVA36_REF | 80.72 |
| MW587071_RVA_S0688LYH | FJ445166_RVA89_F08 | 80.79 |
| AY355188_RVA7_REF | FJ445184_RVA89_ATCC_VR_1199 | 80.79 |
| RV-AZ88**-USA-Nov-2020 | JQ837716_RVA89_p1290_s3853 | 80.84 |
| LC428138_RVA_12_SGH_JPN_2015 | AY355239_RVA58_REF | 80.84 |
| LC428138_RVA_12_SGH_JPN_2015 | KM361528_RVA_CU106 | 80.84 |
| FJ445184_RVA89_ATCC_VR_1199 | KP737114_RVA_F0062 | 80.84 |
| AY355270_RVA89_REF | KP737114_RVA_F0062 | 80.84 |
| AY355188_RVA7_REF | AY355270_RVA89_REF | 80.90 |
| RV-AZ88**-USA-Nov-2020 | JQ837719_RVA89_p1052_s3032 | 80.95 |
| LN623990_RVA_Tk0901_K590 | FJ445165_RVA89_F09 | 80.95 |
| AY355188_RVA7_REF | AY355239_RVA58_REF | 80.95 |
| LN623990_RVA_Tk0901_K590 | JQ837716_RVA89_p1290_s3853 | 81.07 |
| DQ473504_RVA88 | FJ445184_RVA89_ATCC_VR_1199 | 81.07 |
| DQ473504_RVA88 | AY355270_RVA89_REF | 81.07 |
| AY450518_RVA88 | FJ445184_RVA89_ATCC_VR_1199 | 81.07 |
| AY450518_RVA88 | AY355270_RVA89_REF | 81.07 |
| LN623990_RVA_Tk0901_K590 | FJ445166_RVA89_F08 | 81.18 |
| LN623990_RVA_Tk0901_K590 | JQ837719_RVA89_p1052_s3032 | 81.18 |
| AY355269_RVA88_REF | FJ445184_RVA89_ATCC_VR_1199 | 81.18 |
| AY355269_RVA88_REF | AY355270_RVA89_REF | 81.18 |
| AY355269_RVA88_REF | AY355239_RVA58_REF | 81.18 |
| LN623990_RVA_Tk0901_K590 | MW587058_RVA_S0579HDY | 81.30 |
| DQ473504_RVA88 | AY355239_RVA58_REF | 81.30 |
| AY355188_RVA7_REF | MW587058_RVA_S0579HDY | 81.35 |
| LN623990_RVA_Tk0901_K590 | AY355239_RVA58_REF | 81.42 |
| AY450518_RVA88 | AY355239_RVA58_REF | 81.42 |
| AY355188_RVA7_REF | FJ445165_RVA89_F09 | 81.47 |
| RV-AZ88**-USA-Nov-2020 | MW587058_RVA_S0579HDY | 81.53 |
| FJ445166_RVA89_F08 | KP737114_RVA_F0062 | 81.53 |
| AY355188_RVA7_REF | JQ837716_RVA89_p1290_s3853 | 81.56 |
| RV-AZ88**-USA-Nov-2020 | FJ445165_RVA89_F09 | 81.65 |
| AY355188_RVA7_REF | KF034079_RVA_Tk0809_E332 | 81.65 |
| FJ445165_RVA89_F09 | KP737114_RVA_F0062 | 81.65 |
| JQ837716_RVA89_p1290_s3853 | KP737114_RVA_F0062 | 81.65 |
| AY355217_RVA36_REF | KP737114_RVA_F0062 | 81.65 |
| AY355239_RVA58_REF | KP737114_RVA_F0062 | 81.65 |
| AY355188_RVA7_REF | JQ837719_RVA89_p1052_s3032 | 81.68 |
| RV-AZ88**-USA-Nov-2020 | AY355239_RVA58_REF | 81.77 |
| MW587071_RVA_S0688LYH | AY355269_RVA88_REF | 81.77 |
| JQ837719_RVA89_p1052_s3032 | KP737114_RVA_F0062 | 81.77 |
| MW587071_RVA_S0688LYH | DQ473504_RVA88 | 81.88 |
| MW587071_RVA_S0688LYH | AY450518_RVA88 | 81.88 |
| FJ445184_RVA89_ATCC_VR_1199 | KM361528_RVA_CU106 | 82.00 |
| AY355270_RVA89_REF | KM361528_RVA_CU106 | 82.00 |
| AY355188_RVA7_REF | FJ445166_RVA89_F08 | 82.05 |
| RV-AZ88**-USA-Nov-2020 | FJ445166_RVA89_F08 | 82.11 |
| MW587058_RVA_S0579HDY | KP737114_RVA_F0062 | 82.11 |
| FJ445166_RVA89_F08 | KM361528_RVA_CU106 | 82.23 |
| AY355188_RVA7_REF | AY355269_RVA88_REF | 82.35 |
| FJ445165_RVA89_F09 | KM361528_RVA_CU106 | 82.35 |
| JQ837716_RVA89_p1290_s3853 | KM361528_RVA_CU106 | 82.35 |
| AY355239_RVA58_REF | KM361528_RVA_CU106 | 82.35 |
| AY355188_RVA7_REF | DQ473504_RVA88 | 82.46 |
| AY355188_RVA7_REF | AY450518_RVA88 | 82.46 |
| AY355188_RVA7_REF | LC699419_RVA88_Fukushima_H581_2019 | 82.46 |
| JQ837719_RVA89_p1052_s3032 | KM361528_RVA_CU106 | 82.46 |
| RV-AZ88**-USA-Nov-2020 | KF034079_RVA_Tk0809_E332 | 82.58 |
| MW587071_RVA_S0688LYH | KF034079_RVA_Tk0809_E332 | 82.58 |
| MW587071_RVA_S0688LYH | LC699419_RVA88_Fukushima_H581_2019 | 82.58 |
| AY355188_RVA7_REF | LC428139_RVA_21_SGH_JPN_2015 | 82.69 |
| MW587058_RVA_S0579HDY | KM361528_RVA_CU106 | 82.69 |
| AY355217_RVA36_REF | KM361528_RVA_CU106 | 82.81 |
| AY355188_RVA7_REF | LC428138_RVA_12_SGH_JPN_2015 | 83.04 |
| RV-AZ88**-USA-Nov-2020 | LC428139_RVA_21_SGH_JPN_2015 | 83.28 |
| LN623990_RVA_Tk0901_K590 | DQ473504_RVA88 | 83.39 |
| LN623990_RVA_Tk0901_K590 | AY355269_RVA88_REF | 83.39 |
| LN623990_RVA_Tk0901_K590 | AY450518_RVA88 | 83.39 |
| MW587071_RVA_S0688LYH | LC428139_RVA_21_SGH_JPN_2015 | 83.39 |
| RV-AZ88**-USA-Nov-2020 | LC699419_RVA88_Fukushima_H581_2019 | 83.51 |
| RV-AZ88**-USA-Nov-2020 | AY355188_RVA7_REF | 83.62 |
| RV-AZ88**-USA-Nov-2020 | LC428138_RVA_12_SGH_JPN_2015 | 83.62 |
| LN623990_RVA_Tk0901_K590 | AY355188_RVA7_REF | 83.74 |
| RV-AZ88**-USA-Nov-2020 | DQ473504_RVA88 | 83.86 |
| RV-AZ88**-USA-Nov-2020 | AY355269_RVA88_REF | 83.86 |
| RV-AZ88**-USA-Nov-2020 | AY450518_RVA88 | 83.86 |
| LN623990_RVA_Tk0901_K590 | LC699419_RVA88_Fukushima_H581_2019 | 83.97 |
| MW587071_RVA_S0688LYH | LC428138_RVA_12_SGH_JPN_2015 | 83.97 |
| AY355217_RVA36_REF | AY355239_RVA58_REF | 83.97 |
| RV-A88Z**-USA-Nov-2020 | MW587071_RVA_S0688LYH | 84.09 |
| LN623990_RVA_Tk0901_K590 | KF034079_RVA_Tk0809_E332 | 84.55 |
| JQ837716_RVA89_p1290_s3853 | AY355239_RVA58_REF | 84.90 |
| JQ837719_RVA89_p1052_s3032 | AY355239_RVA58_REF | 85.02 |
| LN623990_RVA_Tk0901_K590 | MW587071_RVA_S0688LYH | 85.13 |
| LN623990_RVA_Tk0901_K590 | LC428139_RVA_21_SGH_JPN_2015 | 85.13 |
| FJ445166_RVA89_F08 | AY355239_RVA58_REF | 85.25 |
| FJ445165_RVA89_F09 | AY355239_RVA58_REF | 85.25 |
| MW587058_RVA_S0579HDY | AY355239_RVA58_REF | 85.25 |
| FJ445184_RVA89_ATCC_VR_1199 | AY355239_RVA58_REF | 85.25 |
| AY355270_RVA89_REF | AY355239_RVA58_REF | 85.25 |
| JQ837716_RVA89_p1290_s3853 | AY355217_RVA36_REF | 85.47 |
| LN623990_RVA_Tk0901_K590 | LC428138_RVA_12_SGH_JPN_2015 | 85.48 |
| FJ445165_RVA89_F09 | AY355217_RVA36_REF | 85.58 |
| JQ837719_RVA89_p1052_s3032 | AY355217_RVA36_REF | 85.58 |
| FJ445166_RVA89_F08 | AY355217_RVA36_REF | 86.39 |
| MW587058_RVA_S0579HDY | AY355217_RVA36_REF | 86.39 |
| FJ445184_RVA89_ATCC_VR_1199 | AY355217_RVA36_REF | 87.31 |
| AY355270_RVA89_REF | AY355217_RVA36_REF | 87.31 |
| MW587071_RVA_S0688LYH | AY355188_RVA7_REF | 89.24 |
| AY355269_RVA88_REF | KF034079_RVA_Tk0809_E332 | 90.71 |
| AY450518_RVA88 | KF034079_RVA_Tk0809_E332 | 90.71 |
| DQ473504_RVA88 | KF034079_RVA_Tk0809_E332 | 90.82 |
| AY355269_RVA88_REF | LC699419_RVA88_Fukushima_H581_2019 | 90.82 |
| DQ473504_RVA88 | LC699419_RVA88_Fukushima_H581_2019 | 90.94 |
| AY450518_RVA88 | LC699419_RVA88_Fukushima_H581_2019 | 90.94 |
| AY355269_RVA88_REF | LC428139_RVA_21_SGH_JPN_2015 | 91.17 |
| AY450518_RVA88 | LC428139_RVA_21_SGH_JPN_2015 | 91.17 |
| DQ473504_RVA88 | LC428139_RVA_21_SGH_JPN_2015 | 91.29 |
| AY355269_RVA88_REF | LC428138_RVA_12_SGH_JPN_2015 | 91.29 |
| AY450518_RVA88 | LC428138_RVA_12_SGH_JPN_2015 | 91.29 |
| FJ445165_RVA89_F09 | FJ445184_RVA89_ATCC_VR_1199 | 91.35 |
| FJ445165_RVA89_F09 | AY355270_RVA89_REF | 91.35 |
| DQ473504_RVA88 | LC428138_RVA_12_SGH_JPN_2015 | 91.41 |
| MW587058_RVA_S0579HDY | FJ445184_RVA89_ATCC_VR_1199 | 91.58 |
| MW587058_RVA_S0579HDY | AY355270_RVA89_REF | 91.58 |
| FJ445166_RVA89_F08 | FJ445184_RVA89_ATCC_VR_1199 | 91.81 |
| FJ445166_RVA89_F08 | AY355270_RVA89_REF | 91.81 |
| JQ837716_RVA89_p1290_s3853 | FJ445184_RVA89_ATCC_VR_1199 | 92.50 |
| JQ837716_RVA89_p1290_s3853 | AY355270_RVA89_REF | 92.50 |
| JQ837719_RVA89_p1052_s3032 | FJ445184_RVA89_ATCC_VR_1199 | 92.62 |
| JQ837719_RVA89_p1052_s3032 | AY355270_RVA89_REF | 92.62 |
| RV-AZ88**-USA-Nov-2020 | LN623990_RVA_Tk0901_K590 | 93.26 |
| LC428139_RVA_21_SGH_JPN_2015 | LC699419_RVA88_Fukushima_H581_2019 | 94.89 |
| LC428138_RVA_12_SGH_JPN_2015 | LC699419_RVA88_Fukushima_H581_2019 | 95.01 |
| KF034079_RVA_Tk0809_E332 | LC699419_RVA88_Fukushima_H581_2019 | 95.24 |
| JQ837716_RVA89_p1290_s3853 | MW587058_RVA_S0579HDY | 95.50 |
| JQ837719_RVA89_p1052_s3032 | MW587058_RVA_S0579HDY | 95.62 |
| LC428139_RVA_21_SGH_JPN_2015 | KF034079_RVA_Tk0809_E332 | 96.17 |
| LC428138_RVA_12_SGH_JPN_2015 | KF034079_RVA_Tk0809_E332 | 96.28 |
| KP737114_RVA_F0062 | KM361528_RVA_CU106 | 96.28 |
| FJ445165_RVA89_F09 | MW587058_RVA_S0579HDY | 96.42 |
| FJ445166_RVA89_F08 | MW587058_RVA_S0579HDY | 96.54 |
| FJ445166_RVA89_F08 | JQ837716_RVA89_p1290_s3853 | 96.77 |
| FJ445166_RVA89_F08 | JQ837719_RVA89_p1052_s3032 | 96.89 |
| FJ445165_RVA89_F09 | JQ837716_RVA89_p1290_s3853 | 97.00 |
| FJ445165_RVA89_F09 | JQ837719_RVA89_p1052_s3032 | 97.12 |
| FJ445166_RVA89_F08 | FJ445165_RVA89_F09 | 97.23 |
| LC428138_RVA_12_SGH_JPN_2015 | LC428139_RVA_21_SGH_JPN_2015 | 99.42 |
| AY355269_RVA88_REF | AY450518_RVA88 | 99.77 |
| FJ445184_RVA89_ATCC_VR_1199 | AY355270_RVA89_REF | 99.77 |
| DQ473504_RVA88 | AY355269_RVA88_REF | 99.88 |
| DQ473504_RVA88 | AY450518_RVA88 | 99.88 |
| JQ837719_RVA89_p1052_s3032 | JQ837716_RVA89_p1290_s3853 | 99.88 |

**Figure legends**

**Figure S1**. **Gel electrophoresis result of partial VP1 (~350bp) assay**. Lanes 1 and 8 are 100bp molecular ladder. Gel visualized using BioRad Gel Doc XR+ system running Image lab 4.1 software with option to “highlight saturated pixels” enabled.
