# Supplementary figures and images for: Surveillance of rhinovirus diversity among a university community identifies multiple types from all three species including an unassigned rhinovirus A genotype
Source: Influenza Other Respir Viruses. 2022 Sep 28;17(1):e13057. doi: 10.1111/irv.13057 (PMC9835438; doi:10.1111/irv.13057)

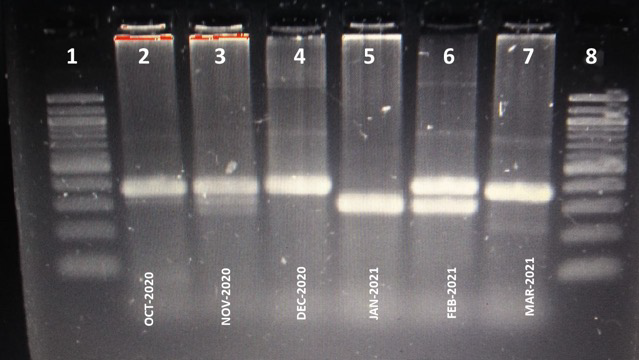

Supplement: Supplementary file 2 — Figure S1. Gel electrophoresis result of partial VP1 (~350 bp) assay. Lanes 1 and 8 are 100 bp molecular ladder. Gel visualized using BioRad Gel Doc XR + system running Image lab 4.1 software with option to “highlight saturated pixels” enabled. [file IRV-17-e13057-s001.tiff]
